# Clinical importance of weight gain and associated factors in patients with moderate to severe ulcerative colitis: results from the MOSAIK cohort in Korea

**DOI:** 10.1186/s12876-023-03008-7

**Published:** 2023-11-21

**Authors:** Hyuk Yoon, Young Soo Park, Jeong Eun Shin, Byong Duk Ye, Chang Soo Eun, Soon Man Yoon, Jae Myung Cha, You Sun Kim, Kyu Chan Huh, Young Sook Park, Jae Hee Cheon, Eun Suk Jung, Youngdoe Kim, Su Young Jung

**Affiliations:** 1 Department of Internal Medicine, Seoul National University Bundang Hospital, Seoul National University College of Medicine, 300 Gumi-Dong, Bundang-Gu, Seongnam, Gyeonggi-Do 463-707 South Korea; 2 Department of Internal Medicine, Dankook University College of Medicine, Cheonan, Korea; 3 Department of Gastroenterology and Inflammatory Bowel Disease Center, Asan Medical Center, University of Ulsan College of Medicine, Seoul, Korea; 4 Department of Internal Medicine, Hanyang University Guri Hospital, Guri, Korea; 5 Department of Internal Medicine, Chungbuk National University Hospital, Chungbuk National University College of Medicine, Cheongju, Korea; 6 Department of Internal Medicine, Kyung Hee University College of Medicine, Kyung Hee University Hospital at Gangdong, Seoul, Korea; 7 Department of Internal Medicine, Seoul Paik Hospital, Inje University College of Medicine, Seoul, Korea; 8 Division of Gastroenterology, Department of Internal Medicine, Konyang University Hospital, Konyang University College of Medicine, Daejeon, Korea; 9 Department of Internal Medicine, Nowon Eulji University School of Medicine, Eulji Hospital, Seoul, Korea; 10 Department of Internal Medicine, Institute of Gastroenterology, Yonsei University College of Medicine, Seoul, Korea; 11 Medical Affairs, Janssen Korea Ltd., Seoul, Korea

**Keywords:** Ulcerative colitis, Weight gain, Risk factors

## Abstract

**Background:**

Many patients with ulcerative colitis (UC) gain weight after treatment. However, the clinical significance of weight gain in these patients remains unclear. This study aimed to evaluate body weight changes after treatment in patients newly diagnosed with moderate-to-severe UC and their effects on patients’ prognosis.

**Methods:**

The change in weight between diagnosis and 1 year after treatment in 212 patients enrolled in the MOSAIK cohort (mean age, 40 years; males, 60%) was analyzed. Significant weight gain was defined as a weight increase of ≥ 5% from the baseline at 1 year. Factors associated with significant weight gain and the effect of significant weight gain on the risk of major adverse outcomes (clinical relapse, hospitalization, and new use of steroids or biologics) during a follow-up period of 20 months were evaluated.

**Results:**

Mean weight gain at 1 year was 1.7 ± 4.2 kg. The proportion of overweight/obese patients increased by 9.0% from 37.9% to 46.9%. Thirty-two percent had significant weight gain; extensive colitis at diagnosis was the only factor associated with significant weight gain (odds ratio 6.5, 95% confidence interval 1.4–31.0, *p* = 0.006). In multivariable analysis, significant weight gain was not associated with the risk of major adverse outcomes. Weight loss symptoms at diagnosis were associated with an increased risk for new steroid use after 1 year.

**Conclusions:**

Approximately one-third of patients with moderate-to-severe UC had significant weight gain after 1 year of treatment. However, significant weight gain was not associated with the patient’s prognosis.

**Supplementary Information:**

The online version contains supplementary material available at 10.1186/s12876-023-03008-7.

## Background

Many studies have investigated the correlation between inflammatory bowel disease (IBD) and obesity. First, several studies have reported that obesity increases the risk of Crohn’s disease but is not related to the risk of ulcerative colitis (UC) [1, 2]. Second, among patients already diagnosed with IBD, obesity generally has adverse effects on the course of IBD [3, 4]. Obesity in patients with IBD has been known to increase the risk of postoperative infection and hospitalization [4, 5]. However, some conflicting results have been reported regarding whether obesity increases the risk of treatment failure [6, 7].

Weight gain is different from obesity. Obesity is an excessive fat accumulation that presents a health risk and is a static condition. In contrast, weight gain is a dynamic condition. Because many patients with IBD have significant weight loss before diagnosis, [8] weight gain after treatment of IBD does not necessarily mean obesity. Although many IBD patients complain of unwanted excess weight gain after treatment, the clinical meaning of weight gain during the treatment of IBD is unclear. Studies on weight gain and its effects on IBD's natural course have been mainly conducted in children [9]. Only a few studies have been performed in adults, usually regarding patients taking anti-TNF agents [10, 11]. A few studies have followed up patients for over a year.

Given this background, first, this study aimed to evaluate changes in body weight over 1 year in patients newly diagnosed with moderate-to-severe UC and to analyze factors associated with significant weight gain. Second, we evaluated whether significant weight gain within 1 year after diagnosis affects adverse clinical outcomes regarding clinical relapse, UC-related hospitalization, and new use of steroids or biologics.

## Methods

### Patients

We analyzed the change in weights between diagnosis and 1 year after treatment in patients enrolled in the MOSAIK cohort. The MOSAIK cohort was a nationwide, multicenter, prospective, hospital-based, observational cohort of patients with newly diagnosed moderate-to-severe UC who were recruited from 30 academic tertiary hospitals in Korea [12]. Patients who satisfied the following criteria were included in the cohort: (1) newly diagnosed with moderate-to-severe UC aged 7 years or older, (2) diagnosed at a tertiary referral hospital within 4 weeks from the visit or diagnosed at a primary or secondary referral hospital with subsequent confirmation by a tertiary center within 8 weeks; and (3) agreed with informed consent. In the MOSAIK cohort study, data on age, sex, height, weight, disease extent at baseline, disease activity at diagnosis and follow-up years, smoking history, extraintestinal manifestations, major clinical outcomes including clinical relapse, UC-related hospitalization, new use of steroids and biologics, and medication use for UC treatment were collected. Among 368 patients who were enrolled in the MOSAIK cohort between August 2014 and February 2017, 156 patients were excluded for the following reasons: not meeting the inclusion criteria (*N* = 14), age < 17 years (*N* = 5), and unavailable data on body weight at baselines and 1 year (*N* = 137). Finally, 212 patients (mean age, 40 years; 60% men) were included in the analysis. This study was conducted in accordance with the Declaration of Helsinki and approved by the institutional review boards of all participating hospitals (Seoul National University Bundang Hospital, approval number: B-1402/240–005). Written informed consent was obtained from all the participants and the data used in this study was anonymized before its use. This study was registered at www.clinicaltrials.gov in 01/09/2014 (ClinicalTrials.gov identifier: NCT02229344).

### Definitions

Body weight data were collected at baseline and 1 year after UC diagnosis. Body weight was classified as underweight (body mass index (BMI) < 18.5 kg/m^2^), normal (18.5 kg/m^2^ ≤ BMI < 23 kg/m^2^), overweight (23 kg/m^2^ ≤ BMI < 25 kg/m^2^), and obese (BMI ≥ 25 kg/m^2^) according to the guideline for the management of obesity by the Korean Society for the Study of Obesity [13]. Significant weight gain (SWG) was defined as a weight increase of ≥ 5% from baseline 1 year after diagnosis, and patients were divided into the SWG and non-SWG groups. We used age, sex, disease extent, disease activity, weight loss, smoking history, extraintestinal manifestations, and initial systemic steroid use as clinical characteristics at diagnosis. We also used disease activity, number of relapses, and exposed medications (systemic steroid, immunomodulators, biologics) as clinical characteristics at 1 year. Disease activity was defined using the partial Mayo Clinic score (PMS) as follows: remission (PMS 0–1), mild (PMS 2–4), moderate (PMS 5–7), and severe (PMS 8–9). Medications administered within 4 weeks of diagnosis were defined as the initial treatment. In addition, we analyzed the potential factors associated with significant weight gain within 1 year after diagnosis and those associated with adverse clinical outcomes, including clinical relapse, UC-related hospitalization, and new use of steroids or biologics. Clinical relapse was defined as an increase in PMS of ≥ 3 points from clinical remission or partial response. Clinical remission was defined as a PMS of ≤ 2 points with no individual sub-score > 1 point, and clinical response was defined as a decrease in PMS of ≥ 2 points and ≥ 30% from baseline PMS, plus either a reduction in the rectal bleeding sub-score of ≥ 1 point or an absolute rectal bleeding sub-score of 0 or 1 [14, 15].

### Statistical analysis

Descriptive statistics for continuous variables were presented as means with standard deviations, and categorical variables were presented as frequencies with percentages in parentheses. To compare the variables between SWG and non-SWG groups, we used the Student’s *t*-test or the Mann–Whitney *U*-test for continuous variables and the Pearson’s Chi-squared test or Fisher’s exact test for categorical variables, where appropriate. Logistic regression analysis was used to assess factors associated with significant weight gain one year after diagnosis. Each factor’s effect on significant weight gain was expressed as an odds ratio (OR) with 95% confidence intervals (CI). Cox proportional hazards model was used to assess the effect of significant weight gain one year after diagnosis on adverse clinical outcomes. The effects of clinical variables on adverse clinical outcomes were expressed as hazard ratios (HRs) with 95% CI. For multivariable logistic regression and Cox proportional hazards analyses, variables with a *p*-value < 0.1 in univariate analysis were included. Age and sex were used as primary covariates in multiple models, regardless of *p*-values. Also, a comparison of adverse clinical outcomes between SWG and non-SWG groups one year after diagnosis was assessed using Kaplan–Meier survival analysis with the log-rank test. We used all observed values for all analyses without any imputations. All analyses were performed using the SAS software (version 9.4; SAS Institute Inc., Cary, NC, USA). Two-sided *p*-values less than 0.05 were considered statistically significant.

## Results

**Fig. 1:**
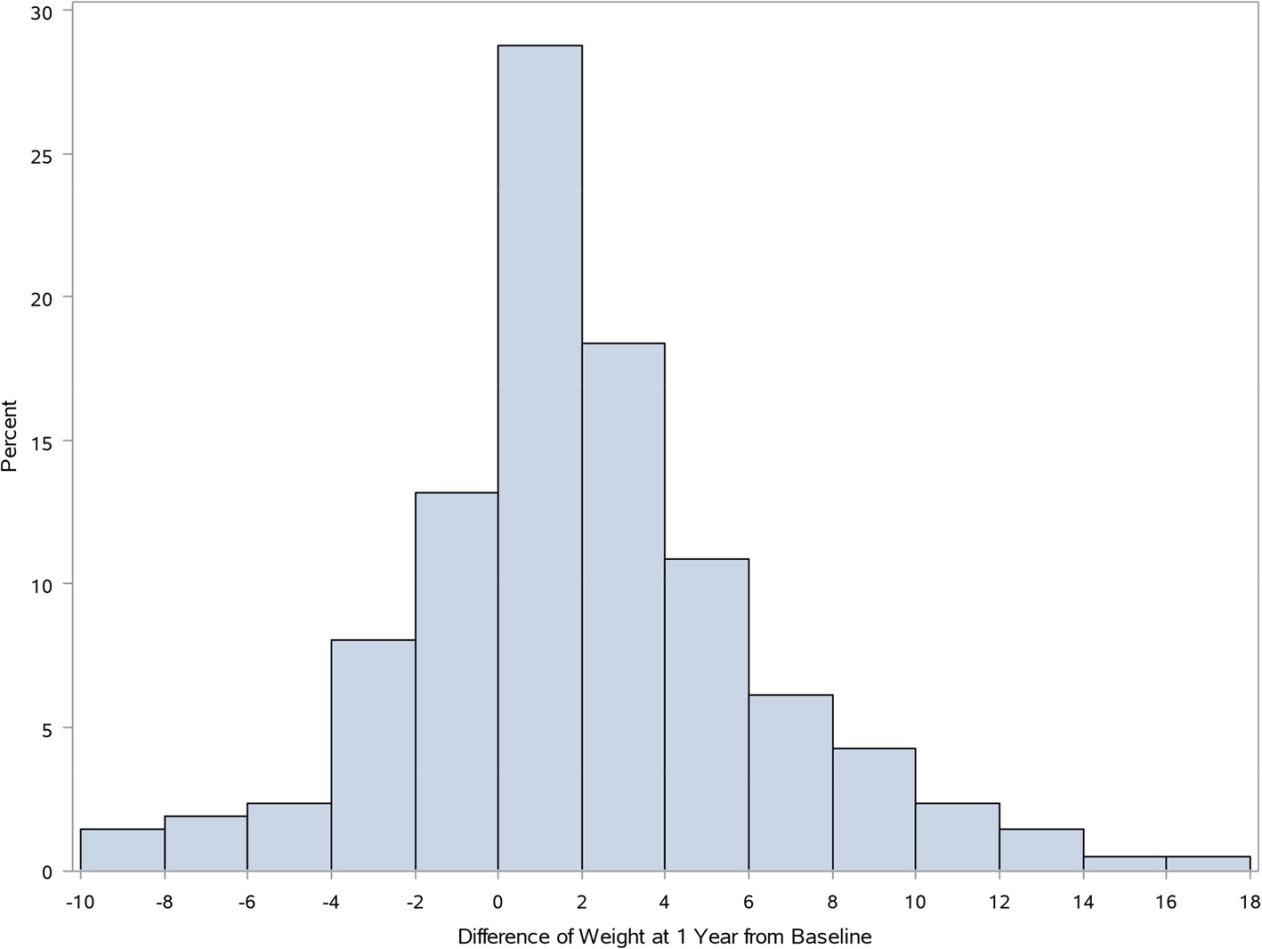
Histogram of weight change in patients with moderate-to-severe ulcerative colitis during 1 year after diagnosis

Figure 1 shows a histogram of the weight changes in the study population 1 year after diagnosis. The mean weight gain in these patients was 1.7 kg (standard deviation, 4.2) at 1 year. Median weight gain was 1.0 kg (range, -9.5–16.0). Table 1 shows the categorized weight changes in the study population 1 year after diagnosis. Thirty-two percent (68/212) had significant weight gain 1 year after diagnosis. The proportion of overweight/obese patients increased by 9.0% from 37.9% to 46.9%. Table 2 shows the clinical characteristics of the study population. Approximately 20% (43/212) of patients experienced weight loss at diagnosis. About 50% (107/212) of the patients were administered steroids as their first treatment. Among the 26 biologics users, only one in the non-SWG group was treated with vedolizumab, and all others were treated with anti-TNF agents. Patients who gained weight had a more extensive disease at diagnosis. In posthoc comparisons, patients in the SWG group had significantly more extensive colitis than those in the non-SWG group (proctitis vs. extensive colitis: *p* = 0.0047, left-sided colitis vs. extensive colitis: *p* = 0.0120). Patients in the SWG group had fewer relapses (0.7 vs. 0.4, *p* < 0.001). In addition, they had lower disease activity at 1 year (remission:52.4% vs. 75%, *p* = 0.013). In multivariable analysis, extensive colitis at diagnosis was the only factor associated with significant weight gain at 1 year (OR 6.5, 95% CI 1.4–31.0, *p* = 0.006) (Table 3).

**Table 1:** Body weight change over 1 year in patients newly diagnosed as moderate-to-severe ulcerative colitis

	At diagnosis				Total (%)
	Underweight	Normal	Overweight	Obese	
At 1 year					
Underweight	9	3	0	0	12 (5.7)
Normal	8	86	5	1	100 (47.4)
Overweight	0	18	26	0	44 (20.9)
Obese	0	7	15	33	55 (26.1)
Total (%)	17 (8.1)	114 (54.0)	46 (21.8)	34 (16.1)	211 (100) ^a^

^a^One patient had missing height at baseline

**Table 2:** Clinical characteristics of the patients with moderate-to-severe ulcerative colitis 1 year after diagnosis

Variables, n (%)	Non-SWG group *N* = 144		SWG group *N* = 68		*P*-value
Age (years), mean (SD)	40.1	(16.14)	38.9	(14.66)	0.720
≤ 40	75	(52.1)	38	(55.9)	0.605
> 40	69	(47.9)	30	(44.1)	
Sex					0.704
Male	85	(59.0)	42	(61.8)	
Female	59	(41.0)	26	(38.2)	
Disease extent at diagnosis					0.003
Proctitis	18	(12.9)	2	(3.0)	
Left-sided colitis	75	(53.6)	27	(40.9)	
Extensive colitis	47	(33.6)	37	(56.1)	
Disease activity at diagnosis^a^					0.168
Moderate	135	(93.8)	60	(88.2)	
Severe	9	(6.3)	8	(11.8)	
Weight loss at diagnosis	27	(18.8)	16	(23.5)	0.419
Smoking history at diagnosis					0.276
Never	71	(50.7)	36	(53.7)	
Past	60	(42.9)	23	(34.3)	
Current	9	(6.4)	8	(11.9)	
EIMs at diagnosis					1.000
No	132	(94.3)	64	(95.5)	
Yes	8	(5.7)	3	(4.5)	
Disease activity at 1 year^b^					0.013
Remission	75	(52.4)	51	(75.0)	
Mild	50	(35.0)	12	(17.6)	
Moderate	16	(11.2)	5	(7.4)	
Severe	2	(1.4)	0	(0.0)	
Number of relapses during the 1 year, mean (SD)	0.7	(0.73)	0.4	(0.71)	< 0.001
Initial systemic steroid use	68	(47.2)	39	(57.4)	0.169
Exposed medication during the 1 year					
Topical and/or systemic 5-ASA	144	(100.0)	68	(100.0)	-
Systemic steroid	90	(62.5)	47	(69.1)	0.347
Immunomodulators	46	(31.9)	22	(32.4)	0.953
Biologics	21	(14.6)	5	(7.4)	0.134

*EIMs* extraintestinal manifestations, *SD* standard deviation, *SWG* significant weight gain, *5-ASA* 5-aminosalisylic acid

^a^Full Mayo clinic score

^b^Partial Mayo clinic score

**Table 3:** Factors associated with significant weight gain over 1 year after diagnosis of moderate-to-severe ulcerative colitis

Variable	Reference	Univariable analysis				Multivariable analysis			
		Odds Ratio	95% CI		*P*-value	Odds Ratio	95% CI		*P*-value
			Lower	Upper			Lower	Upper	
Age group (years) > 40	≤ 40	0.858	0.481	1.532	0.605	0.904	0.481	1.699	0.754
Female	Male	0.892	0.494	1.611	0.704	0.983	0.509	1.898	0.959
Disease extent at diagnosis									
Left-sided colitis	Proctitis	3.240	0.705	14.898	0.661	3.243	0.689	15.255	0.598
Extensive colitis		7.085	1.545	32.494	0.002	6.502	1.363	31.022	0.006
Disease activity at diagnosis^a^									
Severe	Moderate	2.000	0.736	5.436	0.174	-	-	-	-
Weight loss at diagnosis = Yes	No	1.333	0.663	2.683	0.420	-	-	-	-
Smoking history at diagnosis									
Past	Never	0.756	0.404	1.414	0.120	-	-	-	-
Current		1.753	0.624	4.927	0.170	-	-	-	-
EIMs at diagnosis = Yes	No	0.773	0.199	3.014	0.711	-	-	-	-
Disease activity at 1 year = Non-remission^b^	Remission	0.368	0.194	0.697	0.002	0.492	0.227	1.069	0.073
Number of relapses during the 1 year	-	0.525	0.331	0.831	0.006	0.683	0.401	1.164	0.161
Initial systemic steroid use = Yes	No	1.503	0.840	2.688	0.170	-	-	-	-
Exposed medication during the 1 year									
Systemic steroid = Yes	No	1.343	0.726	2.484	0.348	-	-	-	-
Immunomodulators = Yes	No	1.019	0.550	1.889	0.953	-	-	-	-
Biologics = Yes	No	0.465	0.167	1.291	0.142	-	-	-	-

*CI* confidence interval, *EIMs* extraintestinal manifestations

^a^Full Mayo clinic score

^b^Partial Mayo clinic score

**Fig. 2:**
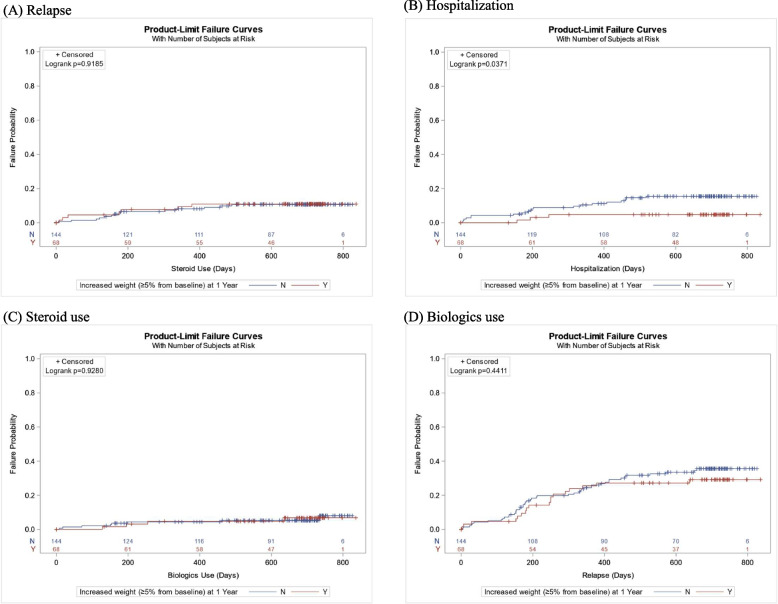
Outcome in patients with moderate-to-severe ulcerative colitis according to weight-gain at 1 year after diagnosis

During the mean 20 months of follow-up 1 year after diagnosis, among major adverse outcomes (clinical relapse, hospitalization, new use of steroids, and biologics), only the risk of hospitalization was lower in patients with significant weight gain than in those without (log-rank test, *p* = 0.037) (Fig. 2). However, significant weight gain 1 year after diagnosis was not associated with the risk of major adverse outcomes in the multivariable analysis (Supplementary Tables S1-4). Only the number of relapses during the first year was associated with both relapse and hospitalization (Supplementary Tables S1 and S2). Weight loss symptoms at diagnosis were associated with an increased risk of new steroid use after 1 year (HR 2.8, 95% CI 1.1–7.0, *p* = 0.026) (Supplementary Table S3). Age over 40, disease activity at 1 year, and exposure to systemic steroids during the first year were associated with an increased risk for new biologic use (Supplementary Table S4).

## Discussion

In this study, 32.1% of patients diagnosed with moderate-to-severe UC had significant weight gain 1 year after diagnosis. In addition, during 1 year of treatment, the proportion of overweight/obese patients increased by 9.0%. According to a study in the United States, the proportion of overweight and obese patients with UC has increased by 2–3 times over the past 40 years [4]. Although no study in Korea has analyzed the trend of obesity focused on IBD patients, it is estimated that the proportion of overweight and obese IBD patients is gradually increasing in IBD patients as in studies conducted on the entire Korean population [16]. The patients with an overweight/obese ratio of 37.9% at diagnosis in this study were not different from the data of the general population with a similar age group in Korea [16]. Because the proportion of obese patients in IBD patients is already not low, a 9% additional change to the overweight/obese group after treatment of UC is considered clinically significant. Since being overweight or obese is a major risk factor for metabolic disorders and adversely affects a patient's overall health, more attention should be paid to this issue.

The proportion of patients who had significant weight gain 1 year after diagnosis (32.1%) was 12% higher than that of patients who lost weight at diagnosis (20.3%). Therefore, a significant proportion of patients have gained more weight. There are several explanations for weight gain in IBD patients. After treatment, an increase in appetite due to improvement in systemic symptoms and subsequent increase in food intake can cause recovery from weight loss and body weight gain. Decreased levels of inflammatory cytokines, which induce catabolism, play a role. Finally, side effects of the drugs used for treating UC are also possible. Approximately half of the patients in this study used systemic steroids as their first treatment, and weight gain is a common adverse effect of steroids. In addition, there are reports that anti-TNF agents also cause weight gain [17]. However, in the multivariate analysis, the use of steroids or anti-TNF agents was not significantly associated with weight gain. Meanwhile, extensive colitis at diagnosis was the only factor associated with significant weight gain at one year, and the OR (6.387) was quite high. Therefore, the degree of initial inflammatory burden and its successful control are more related to weight gain after treatment than the effect of the types of drugs used in patients with moderate-to-severe UC.

In this study, significant weight gain 1 year after diagnosis was not associated with the risk of major adverse outcomes in multivariable analysis. Meanwhile, weight loss symptoms at diagnosis were associated with an increased risk of new steroid use after 1 year. It is not easy to compare with the existing literature because studies with similar designs are lacking. However, unlike obesity at diagnosis in UC patients, weight gain after treatment does not seem to adversely affect the course of the disease [6]. However, since obesity harms overall health and elevated liver enzyme levels due to the development of non-alcoholic fatty liver disease can affect drug use, patients should be informed that maintaining an appropriate weight is essential.

This study has several strengths. We analyzed whether the degree of weight gain one year after diagnosis, not the time of diagnosis, influenced the course of the disease. In addition, this study used prospectively collected data from multiple institutions. However, our study has some limitations. First, since we did not collect data about waist circumference, we could not evaluate abdominal obesity. Visceral adiposity in patients with IBD has been reported to increase the risk of losing response to anti-TNF agents [18]. Second, unlike weight gain, weight loss at the time of diagnosis was analyzed by the patient's subjective answers through a questionnaire without a clear definition. In addition, on the answer sheet, there were only two simple choices, ‘yes’ or ‘no’. Therefore, we could not collect data on how much weight each patient lost. Finally, although we suggest that weight gain after treatment in patients with UC would induce non-alcoholic fatty liver disease because we did not collect data about liver function tests, we cannot confirm this assumption.

In conclusion, approximately one-third of the patients with moderate-to-severe UC had significant weight gain after 1 year of treatment. However, weight gain in these patients was not associated with the risk of major adverse outcomes. Therefore, clinicians may be able to reassure patients with UC that weight gain after treatment does not affect the course of the disease adversely. More research is needed to evaluate the long-term effect of weight gain after treatment for UC on overall health.

### Supplementary Information

**Additional file 1: Supplementary Table S1.** Risk factors for relapse 1 year after diagnosis in patients with moderate-to-severe ulcerative colitis. **Supplementary Table S2.** Risk factors for hospitalization 1 year after diagnosis in patients with moderate-to-severe ulcerative colitis. **Supplementary Table S3.** Risk factors for new use of steroids 1 year after diagnosis in patients with moderate-to-severe ulcerative colitis. **Supplementary Table S4.** Risk factors for new use of biologics 1 year after diagnosis in patients with moderate-to-severe ulcerative colitis.

## Data Availability

The dataset supporting the conclusions of this article is included within the article and its additional file.

## Abbreviations

BMI: Body mass index
CI: Confidence interval
HR: Hazards ratio
IBD: Inflammatory bowel disease
OR: Odds ratio
PMS: Partial Mayo Clinic score
SWG: Significant weight gain
UC: Ulcerative colitis
