# Supplementary material for: Clinical importance of weight gain and associated factors in patients with moderate to severe ulcerative colitis: results from the MOSAIK cohort in Korea
Source: BMC Gastroenterol. 2023 Nov 21;23:405. doi: 10.1186/s12876-023-03008-7 (PMC10664292; doi:10.1186/s12876-023-03008-7)
Supplement: Supplementary file 1 — Additional file 1: Supplementary Table S1. Risk factors for relapse 1 year after diagnosis in patients with moderate-to-severe ulcerative colitis. Supplementary Table S2. Risk factors for hospitalization 1 year after diagnosis in patients with moderate-to-severe ulcerative colitis. Supplementary Table S3. Risk factors for new use of steroids 1 year after diagnosis in patients with moderate-to-severe ulcerative colitis. Supplementary Table S4. Risk factors for new use of biologics 1 year after diagnosis in patients with moderate-to-severe ulcerative colitis. [file 12876_2023_3008_MOESM1_ESM.docx]

**Supplementary Table S1**. Risk factors for relapse 1 year after diagnosis in patients with moderate-to-severe ulcerative colitis

| Variable | Reference | Univariable analysis | | | | | Multivariable analysis | | | |
| --- | --- | --- | --- | --- | --- | --- | --- | --- | --- | --- |
|  |  | Hazard Ratio | 95% CI | | P-value | Hazard Ratio | | 95% CI | | P-value |
|  |  |  | Lower | Upper |  |  |  | Lower | Upper |  |
| Age group (years) > 40 | ≤40 | 0.685 | 0.416 | 1.129 | 0.138 | 0.724 | | 0.436 | 1.204 | 0.214 |
| Female | Male | 0.762 | 0.455 | 1.277 | 0.303 | 0.881 | | 0.524 | 1.484 | 0.635 |
| Significant weight gain at 1 year = Yes* | No | 0.808 | 0.468 | 1.393 | 0.442 | - | | - | - | - |
| Disease extent at diagnosis |  |  |  |  |  |  | |  |  |  |
| Left-sided colitis | Proctitis | 1.287 | 0.540 | 3.066 | 0.569 | - | | - | - | - |
| Extensive colitis |  | 1.060 | 0.432 | 2.604 | 0.899 | - | | - | - | - |
| Disease activity at diagnosis** |  |  |  |  |  |  | |  |  |  |
| Severe | Moderate | 1.483 | 0.676 | 3.251 | 0.326 | - | | - | - | - |
| Weight loss at diagnosis = Yes | No | 1.261 | 0.707 | 2.248 | 0.432 | - | | - | - | - |
| Smoking history at diagnosis |  |  |  |  |  |  | |  |  |  |
| Past | Never | 1.380 | 0.825 | 2.309 | 0.220 | - | | - | - | - |
| Current |  | 0.797 | 0.280 | 2.269 | 0.671 | - | | - | - | - |
| EIMs at diagnosis = Yes | No | 1.103 | 0.400 | 3.038 | 0.850 | - | | - | - | - |
| Disease activity at 1 year = Non-remission*** | Remission | 2.389 | 1.452 | 3.930 | < 0.001 | 1.711 | | 0.973 | 3.009 | 0.062 |
| Number of relapses during the 1 year |  | 1.765 | 1.318 | 2.362 | < 0.001 | 1.487 | | 1.057 | 2.092 | 0.023 |
| Initial systemic steroid use = Yes | No | 0.888 | 0.543 | 1.451 | 0.635 | - | | - | - | - |
| Medication exposed during the 1 year |  |  |  |  |  |  | |  |  |  |
| Systemic steroid = Yes | No | 1.041 | 0.625 | 1.735 | 0.877 | - | | - | - | - |
| Immunomodulators = Yes | No | 1.351 | 0.811 | 2.252 | 0.248 | - | | - | - | - |
| Biologics = Yes | No | 1.535 | 0.782 | 3.015 | 0.213 | - | | - | - | - |

CI: confidence interval, EIMs: extraintestinal manifestations

* Increased weight (>=5% from baseline); **Full Mayo clinic score; *** Not remission by Partial Mayo clinic score

**Supplementary Table S2**. Risk factors for hospitalization 1 year after diagnosis in patients with moderate-to-severe ulcerative colitis

| Variable | Reference | Univariable analysis | | | | Multivariable analysis | | | |
| --- | --- | --- | --- | --- | --- | --- | --- | --- | --- |
|  |  | Hazard Ratio | 95% CI | | P-value | Hazard Ratio | 95% CI | | P-value |
|  |  |  | Lower | Upper |  |  | Lower | Upper |  |
| Age group (years) > 40 | ≤40 | 1.189 | 0.525 | 2.695 | 0.679 | 0.724 | 0.432 | 1.213 | 0.220 |
| Female | Male | 0.999 | 0.432 | 2.309 | 0.999 | 0.872 | 0.516 | 1.473 | 0.609 |
| Significant weight gain at 1 year = Yes* | No | 0.297 | 0.088 | 0.999 | 0.050 | 1.099 | 0.619 | 1.950 | 0.748 |
| Disease extent at diagnosis |  |  |  |  |  |  |  |  |  |
| Left-sided colitis | Proctitis | 0.608 | 0.196 | 1.886 | 0.389 | - | - | - | - |
| Extensive colitis |  | 0.448 | 0.131 | 1.532 | 0.201 | - | - | - | - |
| Disease activity at diagnosis* |  |  |  |  |  |  |  |  |  |
| Severe | Moderate | 1.710 | 0.508 | 5.756 | 0.386 | - | - | - | - |
| Weight loss at diagnosis = Yes | No | 0.565 | 0.168 | 1.902 | 0.357 | - | - | - | - |
| Smoking history at diagnosis |  |  |  |  |  |  |  |  |  |
| Past | Never | 1.784 | 0.752 | 4.235 | 0.189 | - | - | - | - |
| Current |  | 0.647 | 0.082 | 5.106 | 0.679 | - | - | - | - |
| EIMs at diagnosis = Yes | No | 1.687 | 0.393 | 7.242 | 0.482 | - | - | - | - |
| Disease activity at 1 year = Non-remission** | Remission | 4.005 | 1.646 | 9.740 | 0.002 | 1.739 | 0.975 | 3.104 | 0.061 |
| Number of relapses during the 1 year | - | 1.780 | 1.116 | 2.840 | 0.016 | 1.563 | 1.058 | 2.308 | 0.025 |
| Initial systemic steroid use = Yes | No | 0.751 | 0.329 | 1.713 | 0.496 | - | - | - | - |
| Exposed medication during the 1 year |  |  |  |  |  |  |  |  |  |
| Systemic steroid = Yes | No | 2.094 | 0.777 | 5.641 | 0.144 | - | - | - | - |
| Immunomodulators = Yes | No | 3.073 | 1.347 | 7.009 | 0.008 | 0.910 | 0.499 | 1.657 | 0.757 |
| Biologics = Yes | No | 3.577 | 1.470 | 8.703 | 0.005 | 0.895 | 0.409 | 1.960 | 0.781 |

CI: confidence interval, EIMs: extraintestinal manifestations

* Increased weight (>=5% from baseline); **Full Mayo clinic score; *** Not remission by Partial Mayo clinic score

**Supplementary Table S3**. Risk factors for new use of steroids 1 year after diagnosis in patients with moderate-to-severe ulcerative colitis

| Variable | Reference | Univariable analysis | | | | | Multivariable analysis | | | |
| --- | --- | --- | --- | --- | --- | --- | --- | --- | --- | --- |
|  |  | Hazard Ratio | | 95% CI | | P-value | Hazard Ratio | 95% CI | | P-value |
|  |  |  | Lower | | Upper |  |  | Lower | Upper |  |
| Age group (years) > 40 | ≤40 | 0.657 | 0.272 | | 1.585 | 0.350 | 0.755 | 0.307 | 1.859 | 0.541 |
| Female | Male | 0.242 | 0.071 | | 0.821 | 0.023 | 0.314 | 0.090 | 1.098 | 0.070 |
| Significant weight gain at 1 year = Yes* | No | 1.049 | 0.423 | | 2.599 | 0.918 | - | - | - | - |
| Disease extent at diagnosis |  |  |  | |  |  |  |  |  |  |
| Left-sided colitis | Proctitis | 1.962 | 0.249 | | 15.488 | 0.522 | - | - | - | - |
| Extensive colitis |  | 3.018 | 0.390 | | 23.373 | 0.290 | - | - | - | - |
| Disease activity at diagnosis * |  |  |  | |  |  |  |  |  |  |
| Severe | Moderate | 1.993 | 0.587 | | 6.767 | 0.269 | - | - | - | - |
| Weight loss at diagnosis = Yes | No | 2.551 | 1.057 | | 6.154 | 0.037 | 2.823 | 1.135 | 7.020 | 0.026 |
| Smoking history at diagnosis |  |  |  | |  |  |  |  |  |  |
| Past | Never | 0.845 | 0.327 | | 2.179 | 0.727 | - | - | - | - |
| Current |  | 1.682 | 0.469 | | 6.030 | 0.425 | - | - | - | - |
| EIMs at diagnosis = Yes | No | 2.982 | 0.874 | | 10.180 | 0.081 | 2.663 | 0.747 | 9.500 | 0.131 |
| Disease activity at 1 year = Non-remission** | Remission | 1.478 | 0.628 | | 3.481 | 0.371 | - | - | - | - |
| Number of relapses during the 1 year | - | 1.277 | 0.754 | | 2.163 | 0.363 | - | - | - | - |
| Initial systemic steroid use = Yes | No | 1.083 | 0.460 | | 2.550 | 0.855 | - | - | - | - |
| Exposed medication during the 1 year |  |  | | | | |  |  |  |  |
| Systemic steroid = Yes | No | 1.378 | 0.535 | | 3.552 | 0.507 | - | - | - | - |
| Immunomodulators = Yes | No | 1.348 | 0.559 | | 3.253 | 0.506 | - | - | - | - |
| Biologics = Yes | No | 1.755 | 0.590 | | 5.216 | 0.312 | - | - | - | - |

CI: confidence interval, EIMs: extraintestinal manifestations

* Increased weight (>=5% from baseline); **Full Mayo clinic score; *** Not remission by Partial Mayo clinic score

**Supplementary Table S4**. Risk factors for new use of biologics 1 year after diagnosis in patients with moderate-to-severe ulcerative colitis

| Variable | Reference | Univariable analysis | | | | Multivariable analysis | | | |
| --- | --- | --- | --- | --- | --- | --- | --- | --- | --- |
|  |  | Hazard Ratio | 95% CI | | P-value | Hazard Ratio | 95% CI | | P-value |
|  |  |  | Lower | Upper |  |  | Lower | Upper |  |
| Age group (years) > 40 | ≤40 | 5.377 | 1.177 | 24.557 | 0.030 | 7.194 | 1.338 | 38.682 | 0.022 |
| Female | Male | 0.309 | 0.068 | 1.410 | 0.129 | 0.877 | 0.138 | 5.586 | 0.889 |
| Significant weight gain at 1 year = Yes* | No | 1.057 | 0.318 | 3.512 | 0.928 | - | - | - | - |
| Disease extent at diagnosis |  |  |  |  |  |  |  |  |  |
| Left-sided colitis | Proctitis | 0.410 | 0.075 | 2.238 | 0.303 | - | - | - | - |
| Extensive colitis |  | 0.816 | 0.165 | 4.043 | 0.803 | - | - | - | - |
| Disease activity at diagnosis* |  |  |  |  |  |  |  |  |  |
| Severe | Moderate | 0.996 | 0.129 | 7.718 | 0.997 | - | - | - | - |
| Weight loss at diagnosis = Yes | No | 2.091 | 0.629 | 6.954 | 0.229 | - | - | - | - |
| Smoking history at diagnosis |  |  |  |  |  |  |  |  |  |
| Past | Never | 6.146 | 1.328 | 28.448 | 0.020 | 3.729 | 0.562 | 24.719 | 0.173 |
| Current |  | 3.001 | 0.272 | 33.103 | 0.370 | 11.531 | 0.629 | 211.385 | 0.099 |
| EIMs at diagnosis = Yes | No | 3.259 | 0.713 | 14.890 | 0.128 | - | - | - | - |
| Disease activity at 1 year = Non-remission** | Remission | 8.485 | 1.857 | 38.769 | 0.006 | 12.447 | 2.172 | 71.331 | 0.005 |
| Number of relapses during the 1 year | - | 1.864 | 1.011 | 3.438 | 0.046 | 0.619 | 0.257 | 1.493 | 0.286 |
| Initial systemic steroid use = Yes | No | 1.425 | 0.452 | 4.492 | 0.545 | - | - | - | - |
| Exposed medication during the 1 year |  |  |  |  |  |  |  |  |  |
| Systemic steroid = Yes | No | 6.500 | 0.839 | 50.370 | 0.073 | 10.897 | 1.102 | 107.765 | 0.041 |
| Immunomodulators = Yes | No | 3.148 | 0.999 | 9.918 | 0.050 | 1.473 | 0.393 | 5.517 | 0.565 |
| Biologics = Yes | No | 0.660 | 0.085 | 5.114 | 0.691 | - | - | - | - |

CI: confidence interval, EIMs: extraintestinal manifestations

* Increased weight (>=5% from baseline); **Full Mayo clinic score; *** Not remission by Partial Mayo clinic score
